# Impact of *in-utero* electronic cigarette exposure on neonatal neuroinflammation, oxidative stress and mitochondrial function

**DOI:** 10.3389/fphar.2023.1227145

**Published:** 2023-08-24

**Authors:** Sabrina Rahman Archie, Ali Ehsan Sifat, David Mara, Yeseul Ahn, Khondker Ayesha Akter, Yong Zhang, Luca Cucullo, Thomas J. Abbruscato

**Affiliations:** ^1^ Department of Pharmaceutical Sciences, Texas Tech University Health Sciences Center School of Pharmacy, Amarillo, TX, United States; ^2^ Department of Foundation Medical Studies, Oakland University William Beaumont School of Medicine, Rochester, MI, United States

**Keywords:** maternal, neonatal, electronic cigarette, oxidative stress and inflammation, mitochondrial dysfunction, cytokines

## Abstract

**Introduction:** Despite the prevalence of the perception that electronic cigarettes (e-cig) are a safer alternative to tobacco smoke, growing concern about their potential toxic impact warrants adequate investigation focusing on special populations like maternal and pediatric groups. This study evaluated the consequences of maternal e-cig use on neonatal neuroinflammation, oxidative stress, and mitochondrial function in primary cultured neurons and postnatal day (PD) 7 and 90 brain.

**Methodology:** Pregnant CD1 mice were exposed to e‐cig vapor (2.4% nicotine) from gestational day 5 (E5) till PD7, and the primary neurons were isolated from pups at E16/17. Cellular total reactive oxygen species (ROS) and mitochondrial superoxide were measured in primary neurons using CM-H_2_DCFDA and Mitosox red, respectively. Mitochondrial function was assessed by Seahorse XF Cell Mitostress analysis. The level of pro-inflammatory cytokines was measured in primary neurons and PD7 and PD90 brains by RT-PCR and immunobead assay. Western blot analysis evaluated the expression of antioxidative markers (SOD-2, HO-1, NRF2, NQO1) and that of the proinflammatory modulator NF-κB.

**Results:** Significantly higher level of total cellular ROS (*p* < 0.05) and mitochondrial superoxide (*p* < 0.01) was observed in prenatally e-cig-exposed primary neurons. We also observed significantly reduced antioxidative marker expression and increased proinflammatory modulator and cytokines expression in primary neurons and PD7 (*p* < 0.05) but not in PD90 postnatal brain.

**Conclusion:** Our findings suggest that prenatal e-cig exposure induces postnatal neuroinflammation by promoting oxidative stress (OS), increasing cytokines’ levels, and disrupting mitochondrial function. These damaging events can alter the fetal brain’s immune functions, making such offspring more vulnerable to brain insults.

## Introduction

Electronic cigarettes, or e-cigarettes (e-cig) were first introduced in the US market more than a decade ago. E-cig use is commonly known as vaping which has become popular among all age groups and sexes, including women of childbearing age due to its perception as a safer substitute to traditional cigarettes (Archie et al., 2023). According to the latest data, e-cig use is increasingly prevalent among pregnant women, ranging between 5% and 15% of the surveyed pregnant population within the last 5 years (Aboaziza et al., 2022). Pregnant women with a history of conventional tobacco use are switching to e-cig products for fear of poor fetal outcomes from tobacco smoking, including but not limited to abortions and stillbirths, pre-term birth, low birth weight (LBW), intrauterine growth restriction, sudden infant death syndrome (SIDS), neurological and cognitive delays, congenital disabilities, colic, asthma, and ectopic pregnancies (U.S. Department of Health and Human Services, 2014; Archie et al., 2023; Aboaziza et al., 2022; Gould et al., 2020). Nicotine can cross the placenta and decrease uterine blood flow by up to 38% as a vasoconstrictor, leading to fetal oxygen and nutrient deprivation which may ultimately result in hypoxia and malnutrition (Ganapathy et al., 1999; Bush et al., 2000).

Unfortunately, only limited preclinical and clinical studies have been performed to evaluate the safety of either nicotine-free or nicotine-containing e-cig products in pregnancy. Still, several recent emerging reports have suggested that maternal vaping is associated with poor neurodevelopmental outcomes, including LBW, disrupted blood-brain barrier (BBB) integrity, deteriorated motor, learning, and memory function (Archie et al., 2023), decreased glucose utilization, worsened hypoxic-ischemic injury in the postnatal brain (Sifat et al., 2020). In addition, arterial dysfunction (Aboaziza et al., 2022), altered lung development, remodeling and myogenesis (Noël et al., 2020; Wang et al., 2020), alterations in lung transcriptome and increased susceptibility to asthma (Noël et al., 2023), liver dysfunction (Li et al., 2020), renal dysfunction (Li et al., 2019a), cardiovascular deficits (Aboaziza et al., 2022) and epigenetic alterations (Chen et al., 2018a; Nguyen et al., 2018) have also been reported. A recently published work by our group clearly shows that maternal exposure to e-cig aerosol during a gestational period promotes long-term impairment of BBB integrity in the offspring, evidenced by the downregulation of tight junction proteins (TJs) in neonatal mice, which persisted further in neonatal, adolescence and adulthood in a rodent model. Moreover, sex-specific deteriorated motor activity and short-term memory function have also been observed in prenatally e-cig-exposed adolescent and adult mice offspring. Interestingly, our study also identified a sex-difference effect of maternal vaping on the neonatal brain suggesting a potential neuroprotective role for the estrous cycle (Archie et al., 2023). Thus, the current bulk of data strongly suggests a potentially harmful impact of maternal vaping on neonatal health.

Oxidative stress (OS) is essential to the general inflammatory response of the body (Gill et al., 2010). It occurs due to a metabolic redox imbalance caused by the excessive production of reactive oxygen species (ROS) and/or impaired/reduced activity of the cellular antioxidative response mechanisms. Mitochondria are a major site of ROS production during oxidative phosphorylation (OXPHOS) to generate ATP and are thought to increase intracellular OS (Andreyev et al., 2005). During an inflammatory response, a high oxygen level is consumed, and the mitochondria release superoxide free radicals (Allen and Bayraktutan, 2009). The resulting OS can then impair mitochondrial function (Cano et al., 2014), leading to cell and tissue/organ damage. Under normal conditions, the ROS excess is eliminated by antioxidative enzymes, including superoxide dismutase (SOD1, SOD2), glutathione peroxidase (GPX1), heme oxygenase-1 (HO1), catalase (CAT), glutathione-s-transferase (GST).

Nuclear factor (erythroid-derived 2)-like 2 (NRF2) is a transcription factor playing a key role in the activation of the antioxidative response system (by interaction with the antioxidant response element—ARE—sequence in the nuclear DNA) promoting the gene transcription of cytoprotective and antioxidative enzymes such as NAD(P)H quinone oxidoreductase 1 (NQO1), SOD, CAT, GST, HO-1, etc. thus protecting the cell from ROS and electrophilic insults (Dinkova-Kostova et al., 2005; Taguchi et al., 2011; Helou et al., 2019).

NF-κB, instead, is a master pro-inflammatory transcription factor and a pivotal mediator of inflammatory responses. Studies have shown the existence of a crosstalk between NRF2 and NF-κb expression and activity. NRF2 suppresses the activation of the NF-κB pathway by activating/upregulating the antioxidant defense mechanisms, thus reducing ROS-mediated NF-κB activation. By contrast, NF-κB can suppress NRF2 activity and prevent ARE gene transcription (Helou et al., 2019).

Studies have demonstrated that chronic exposure to several exogenous factors, including TS and nicotine, can hamper the activation of the NRF2-ARE signaling pathway and related downstream effectors leading to OS and inflammation (Sivandzade et al., 2019b). In fact, several studies have reported that *in utero* exposure to TS resulted in postnatal inflammation, OS and disrupted neuroimmune and mitochondrial functions (Bruin et al., 2010; Primo et al., 2013; Banderali et al., 2015; Chahal et al., 2017; Cheemarla et al., 2019; Janbazacyabar et al., 2021; Ji et al., 2021; Huang et al., 2022). Interestingly, e-cig exposure has also been identified as a contributor to ROS generation, OS, and neuroinflammation (Chatterjee et al., 2019; Ma et al., 2021; Sun et al., 2021; Emma et al., 2022). Since vaping practice is increasing among women during pregnancy (Whittington et al., 2018) and chronic exposure to e-cig vape is also associated with increased neuroinflammation, like maternal smoking, maternal vaping could also alter the neonatal neuroimmune function and mitochondrial activity, thus making the offspring more vulnerable to brain insults including cerebrovascular and neurodegenerative dysfunction. However, the long-term health effects of maternal vaping on neonatal neuroimmune and mitochondrial function remain unexplored, and no studies have been reported yet to help fill this critical knowledge gap. Hence, this study aims to evaluate the long-term effect of *in utero* e-cig exposures on neuroinflammation, mitochondrial function, and antioxidant capacity of the developing mouse brain and adolescent and adult offspring susceptibility to brain insults.

## Materials and methods

### Animals and surgical procedures

**FIGURE 1:**
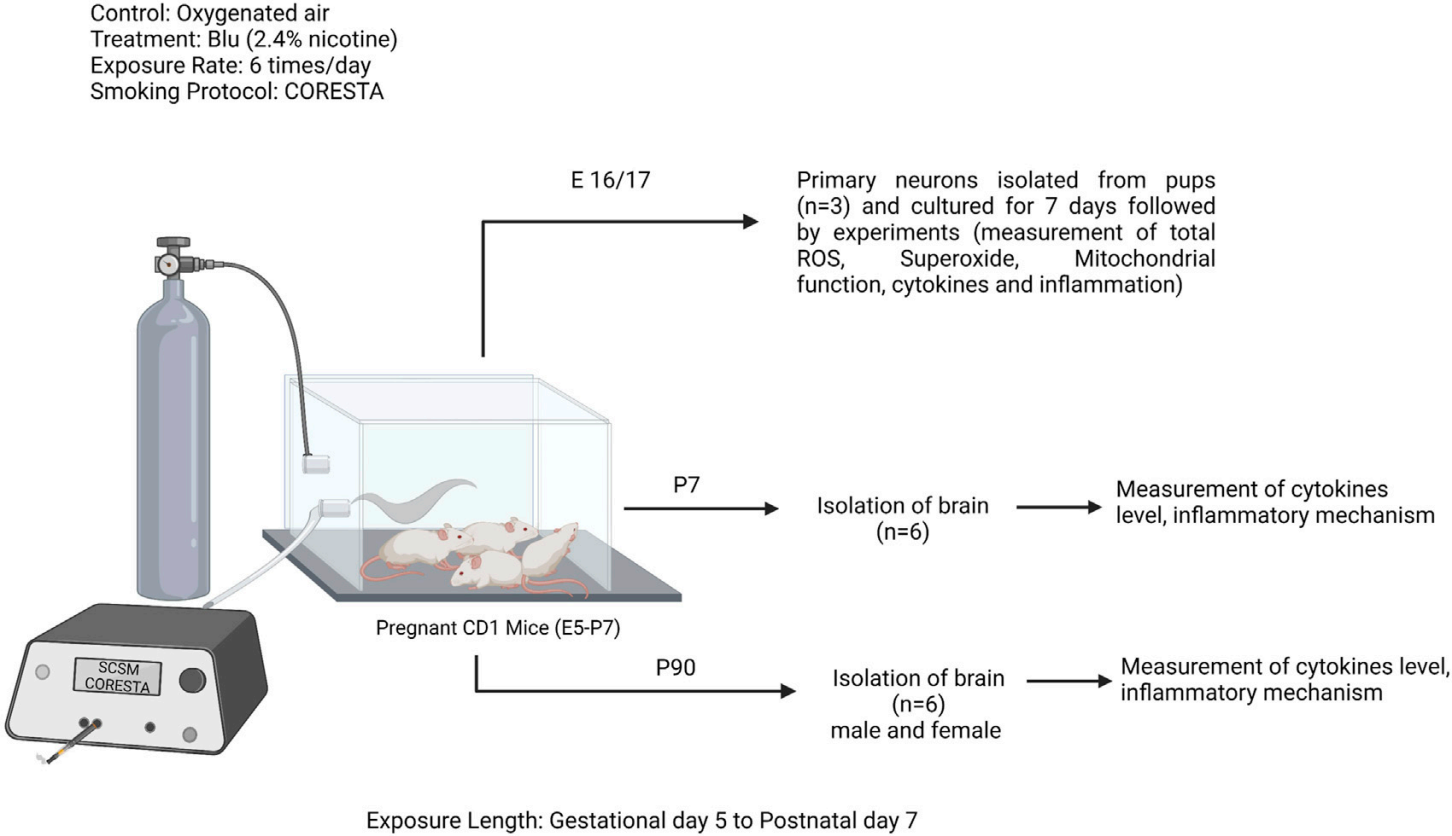
Study design: pregnant CD1 mice were exposed to e-cig (Blu, 2.4% nicotine) from gestational day 5 (E5) to postnatal day 7 (PD7), 6 times per day following the smoking protocol CORESTA. In between this exposure length, a group of pregnant mice (*n* = 3) were sacrificed to isolate the primary neurons from the pups. Rest of the pregnant moms were continued to exposure till PD7. At PD7, another group of offspring were sacrificed (*n* = 10) and isolated the brain. Rest of the offspring were continued to grow till PD90 and another group of both male and female offspring were sacrificed and brains were isolated. Experiments were performed in primary neuron and brain tissue lysate at PD7 and PD90.

All studies were approved by the IACUC of Texas Tech University Health Sciences Center, Lubbock, Texas (IACUC protocol# 20026). Experiments were performed following relevant guidelines and regulations. Female CD1 pregnant mice (Charles River Laboratories, Inc., Wilmington, MA; Cat# CRL:22, RRID: IMSR_CRL:22) were kept under standardized light and dark conditions (12 h), humidity (70%) and temperature (22°C) after delivery their offspring. Pregnant mice were singly housed. Offspring were separated into males and females after weaning (postnatal days 21–22) and housed in a group of 2–5. They were given *ad libitum* access to food and water. Animal behavior was monitored daily to minimize animal suffering. We applied the following exclusion criteria to our experiments: severe weight loss, infections, or significant behavioral deficits (decreased mobility, seizures, lethargy). No animal was excluded from this study. The research design is depicted in Figure 1. A total number of 68 mice were used to perform this study. All experiments were conducted in compliance with the ARRIVE guidelines.

### *In-vivo* e-cig vaping

Pregnant CD1 mice were exposed (via direct inhalation) to e-cig vapor containing 2.4% nicotine (Blu™, 24 mg/mL nicotine) mixed with oxygenated air or oxygenated air alone, six times/day; 2 cartridges/day from gestational day 5 (E5) until delivery. This exposure was continued after delivery until the pups were 7 days old (PD7). The exposure was continued till PD7 as PD7-10 is considered as the peak brain growth spurt period (Semple et al., 2013). After birth, the pups were exposed to nicotine via lactation (Primo et al., 2013; Chan et al., 2016). This exposure model was adopted following a study by Sifat et al., who investigated the effects of prenatal electronic cigarette exposure on offspring in mice models (Li et al., 2012; Sifat et al., 2020). In our study, Blu™ e-cigs were utilized since this is one of the most popular brands still on the market, and there have been previously reported studies using Blu™ (Kaisar et al., 2017; Sifat et al., 2018; Sifat et al., 2020). A modified CORESTA (Cooperation Centre for Scientific Research Relative to Tobacco) standard smoking protocol adapted to study e-cig exposure (27.5 mL puff depth volume, 3 s puff duration, 2 puffs per 60 s, 32 puffs/session) was followed in the laboratory. E-cig vapor was generated using a Single Cigarette Smoking Machines (SCSM, CH Technologies Inc.) following a previously published method used by our laboratory (Kaisar et al., 2017; Sifat et al., 2018; Sifat et al., 2020). This method was developed to mimic the smoking behavior of a human chronic/heavy smoker/vaper and yields plasma levels of cotinine (111 ng/mL) which is in the range of blood cotinine level (30–250 ng/mL) found in other preclinical rodent models of chronic e-cig exposure (McGrath-Morrow et al., 2015; Shi et al., 2019). The smoking exposure was done between 9 a.m. to 2 p.m.

### Primary cortical neuron isolation and culture

Mouse primary cortical neurons were isolated and cultured as previously described (Islam et al., 2016) with a slight modification. In brief, cerebral cortices (pooled from all fetal brains) were isolated from E16 or E17 embryos of control or e-cig-exposed mice and dissected in Hank’s balanced salt solution (HBSS) without Ca2+ and Mg2+ supplemented with 250 μg/mL gentamycin (Fisher Scientific, Hampton, NH; Cat# MT30005CR). 0.25% trypsin was used for 15 min at 37°C followed by neutralization with 10% Fetal Bovine Serum (FBS) to digest the dissected pieces of meninges-free cortices. Dissociated cell suspensions were seeded (1.0 × 10^5 per cm^2 surface area) into poly-D-lysine (Sigma-Aldrich; Cat# P6407) coated 100 mm petri dish, 6- or 96-well plates, seahorse plates, 35 mm glass bottom microwell dishes and cultured in Neurobasal medium (Thermo Fisher Scientific; Cat# A3582901) supplemented with 0.5 mM Glutamax (Thermo Fisher; Cat# 35050061), 25 μg/mL gentamicin, and 2% B27 (Thermo Fisher; Cat# 17504044) at 37°C in a humidified atmosphere of 5% CO2 in air. The medium was replaced with fresh Neurobasal medium after overnight incubation, with half of the media being refreshed every 2 days.

### Measurement of mitochondrial superoxide

For measuring the mitochondrial superoxide through confocal imaging, cells were seeded at a density of 1.5 × 10^5^ cells/cm^2^ on a 35 mm glass bottom petri dish. After 7 days of culture, mitochondrial superoxide was measured by a mitosox red indicator (Thermofisher reagent, catalog). Briefly, stock solution and working solution of mitosox red (5 mM and 1 uM) and mitotracker green (200 uM and 100 nm) were prepared in HBSS according to the manufacturer protocol. After washing three times with prewarmed HBSS, the probe working solution was added to each culture plate. The cultures were incubated at 37°C for 30 min, covered with aluminum foil, and protected from light exposure. After 30 min incubation, the culture plates were washed 3 times with prewarmed HBSS, and confocal images were acquired using a Leica Stellaris SP8 Falcon microscope (Leica Microsystems), and the images (20X magnitude) were captured with the same microscope. The mean total fluorescence intensity was calculated for each color channel, green color (mitochondrial density) and red color (mitochondrial superoxide). The mean intensity of mitochondrial superoxide (mitosox red) was normalized with mitochondrial density (mitotracker green). The images were acquired using identical parameters (smart gain, intensity) across different cultures. Excitation and emission wavelength are as follows. Mitotracker Green: excitation/emission 490/516; Mitosox Red: excitation/emission 396/580.

### Measurement of total reactive oxygen species (ROS)

Total ROS was measured using CM-H_2_DCFDA as a probe. In brief, the cells were plated at a density of 1.5 × 10^5^ cells/cm^2^ on 35 mm glass bottom petri dish, and after 7 days of culture, total ROS was quantified by staining the cultures with 10 uM CM-H_2_DCFDA working solution in HBSS. The cultures were incubated at 37°C for 30 min and covered with aluminum foil to protect them from light. After 30 min incubation, the culture plates were washed 3 times with prewarmed HBSS, and confocal images were acquired using a Leica Stellaris SP8 Falcon microscope (Leica Microsystems) and the images (20X magnitude) were captured with the same microscope at an excitation wavelength of 485 nm and an emission wavelength of 530 nm. The images were acquired using identical parameters (smart gain, intensity) for all the different cultures. The mean intensity of the cellular ROS probe (CM-H_2_DCFDA) was normalized with protein concentration.

Fluorescent intensity was also measured using a microplate reader (BioTek Synergy Mx microplate reader). After taking images, cell lysate was collected using radioimmunoprecipitation assay (RIPA) buffer and centrifugation at 21,000 g for 10 min at 4°C. 100 uL of supernatant was transferred to a black 96 well plate, and fluorescence intensity was measured using a fluorescence microplate reader at an excitation wavelength of 485 nm and an emission wavelength of 530 nm. Finally, fluorescence intensity was normalized with protein concentration.

### Mitochondrial metabolic analysis

Mitochondrial bioenergetic characteristics of primary neurons were determined in an XF24 extracellular flux analyzer (Seahorse Biosciences, Agilent Technologies, Santa Clara, CA) at a density of 1.5 × 10^5^ cells/cm^2^ in 24-well assay plates (Seahorse Bioscience-# 100850- 001). On day 7, the cell medium was replaced by an assay medium consisting of XF Base Medium supplemented with 10 mM glucose, 10 mM pyruvic acid, and 1 mM L-glutamine incubated in a CO_2_-free incubator 1 h before the experiment. Subsequently, the oxygen consumption rate (OCR) analysis was performed in a Seahorse Bioscience XFe24 flux analyzer according to the manufacturer’s instructions. Oligomycin, FCCP (carbonyl cyanide-p trifluoromethoxy phenylhydrazone), and antimycin A/rotenone from a Seahorse XF Cell Mito Stress Test Kit were prepared in an XF assay medium with final concentrations of 1.5 μM, 1.0 μM, and 0.5 μM, respectively, and were serially injected to measure the OCRs of cells in an XF24 plate. After the experiment, cells were lysed using RIPA buffer (ThermoFisher), and proteins were quantified via BCA assay. Results were normalized to the protein concentration of each well to its OCR value.

### Quantitative real-time PCR

Total RNA was extracted from primary neurons isolated from E16/17 as described previously (Akter et al., 2017) using the RNeasy mini kit (Qiagen, Hilden, Germany). A total of 1 µg of RNA was used to synthesize cDNAs using iscript TM Reverse Transcription Supermix (Cat # 170-8841, Bio-Rad, United States). PCR was performed using the iTaq Universal SYBR Green Supermix (Cat # 1725121, Bio-Rad, United States), and the Thermal Cycler CFX96 TM Real-Time System (BIO-RAD, United States) was used for analysis and quantification. The relative mRNA level was normalized to GAPDH, and fold change between control and treated groups was determined using the 2 -δδCt method. The primer pairs were purchased from Integrated DNA Technologies (IDT). The sequences of primers used to amplify each gene have been shown in Table 1. All the primers are from mouse source.

**TABLE 1:** Primer sequences for IL-1β, TNF-α, IL-6, IL-4, IL-10 and GAPDH.

Target mRNA	Forward primer	Reverse primer
IL-1β	5′- CCT​GCA​GCT​GGA​GAG​TGT​GGA​T-3′	5′-TGT​GCT​CTG​CTT​GTG​AGG​TGC​T-3′
TNF-α	5′-AGC​CCA​CGT​CGT​AGC​AAA​CCA​C-3′	5′-AGG​TAC​AAC​CCA​TCG​GCT​GGC​A-3′
IL-6	5′-ACT​TCA​CAA​GTC​GGA​GGC​TT-3′	5′-TGCAAGTGCAT CATCGTTGT-3′
IL-4	5′- TGG​GTC​TCA​ACC​CCC​AGC​TAG​T-3′	TGC​ATG​GCG​TCC​CTT​CTC​CTG​T-3′
IL-10	5′-CAG​TAC​AGC​CGG​GAA​GAC​AA-3′	5′-CCT​GGG​GCA​TCA​CTT​CTA​CC-3′
GAPDH	5′- ACTCACGGCTTCAACG-3′	5′- CCC​TGT​TGC​TGT​AGC​CGT​A- 3′

### Immunobead assay

Immunobead assay was performed to measure the cytokines level in the offspring brain at PD7 and PD90. At the end of the experiments, mouse brain tissues at PD7 and PD90 were homogenized in RIPA buffer supplemented with proteinase inhibitors. The tissue lysate was used for inflammatory cytokines assays with the Mouse High Sensitivity T-Cell 18-plex Discovery Assay^®^ Array (Eve Technologies, Canada). Protein concentrations were used to normalize the data.

### Western blot

Control or e-cig exposed primary cortical neurons were lysed using RIPA buffer to isolate protein lysate. Prenatally e-cig exposed or control mice were sacrificed during neuron isolation at PD7 and PD90. Brains were homogenized and lysed using RIPA buffer to isolate protein lysate. Lysate protein concentration from cell and tissue was determined using a bicinchoninic acid (BCA) assay. Exactly 30 μg of protein from each sample was loaded and separated using a 10% Tris-glycine polyacrylamide precast gel (Bio-Rad Laboratories, Hercules, CA; Cat# 4568034). This method has been used previously to analyze Western blot immunoreactivity (Sifat et al., 2020; Archie et al., 2023). Protein samples were then transferred to a polyvinylidene difluoride membrane (Thermo Fisher; Cat# IPVH00010), and then membranes were incubated in blocking buffer (0.2% Tween-20 containing Tris-buffered saline (TBST) with 5% bovine serum albumin) to block the nonspecific protein bands for 2 h at room temperature. Membranes were incubated with rabbit polyclonal anti-NRF2 antibody (1: 2000, Invitrogen; Cat# 12721), mouse monoclonal anti-NQO1 antibody (1: 1000, Santa Cruz; Cat# SC-376023), rabbit monoclonal anti-SOD2 antibody (1: 2000, Cell Signaling; Cat# D3X8F), rabbit monoclonal anti-HO1 antibody (1:1000, Cell Signaling; Cat# E6Z5G), rabbit monoclonal anti- NF-κB antibody (1:1000, Cell Signaling; Cat# D14E12), rabbit monoclonal anti-Synaptophysin antibody (1: 1000, Cell Signaling; Cat# D8F6H), Rabbit monoclonal anti-PSD95 antibody (1: 1000, Cell Signaling; Cat# D27E11), Rabbit monoclonal antibody anti-Enolase-2 (1:2000, Cell Signaling; Cat# 9536) and mouse monoclonal anti-beta-actin (β-actin) antibody (1: 10000 MilliporeSigma; Cat# A5441) in TBST with 5% bovine serum albumin at 4°C overnight. After 4 times washing with TBST for 15 min each cycle, membranes were incubated with anti-rabbit (Sigma Aldrich; Cat# GENA934- 1ML, RRID: AB_2722659) or anti-mouse (Sigma Aldrich; Cat# GENXA931-1ML, RRID: AB_772209) IgG-horseradish peroxidase secondary antibody (1:10000) in TBST with 5% bovine serum albumin for 2 h at room temperature. After 4 times of 15 min wash with TBST, the protein signals were detected by enhanced chemiluminescence-detecting reagents (Thermo Fisher; Cat# 34577) and visualized in X-ray films in the dark. The protein bands were quantified relative to beta-actin in ImageJ software. The antibody specificity was confirmed by the presence of a single band at Western blotting at the expected molecular weight.

### Body weight measurement

The body weight of both prenatally e-cig exposed offspring and control offspring was measured at PD7 and PD90.

### Statistical analysis

The test for normality was performed to select the appropriate statistical method. All data are expressed as the mean ± SEM. The values were analyzed by unpaired ‘t’ test to compare between two groups (Prism, version 7.0; GraphPad Software Inc., San Diego, CA). *p* values less than 0.05 were considered statistically significant.

## Results

### *In-utero* e-cig exposure increases total cellular ROS and mitochondrial superoxide in primary neurons

**FIGURE 2:**
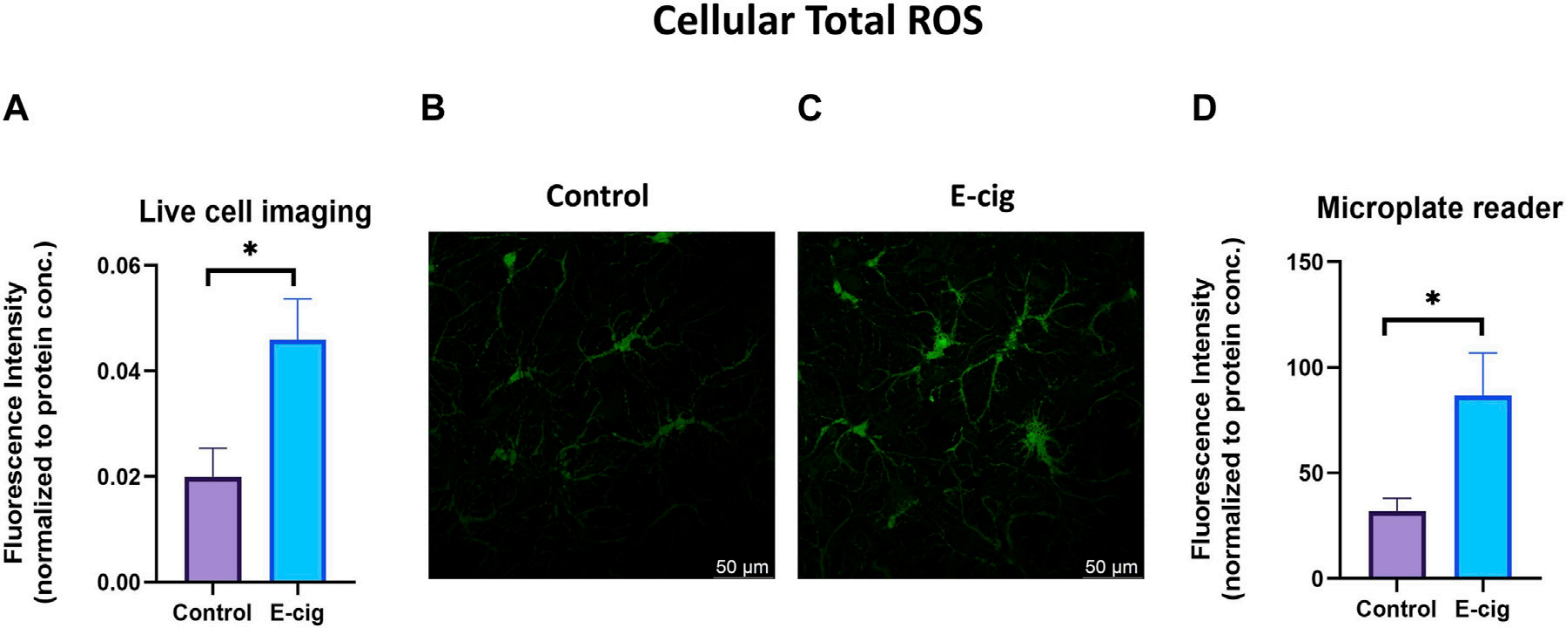
Cellular total ROS was quantified in primary neuron using confocal imaging and microplate reader using CM-H2DCFDA as a probe. Prenatally e-cig exposed primary neuron demonstrated significantly higher level of total ROS (*p* < 0.05) in live cell imaging **(A–C)** and microplate reader **(D)**. Biological replicates, *n* = 3. Fluorescence intensity was normalized by protein concentration.

**FIGURE 3:**
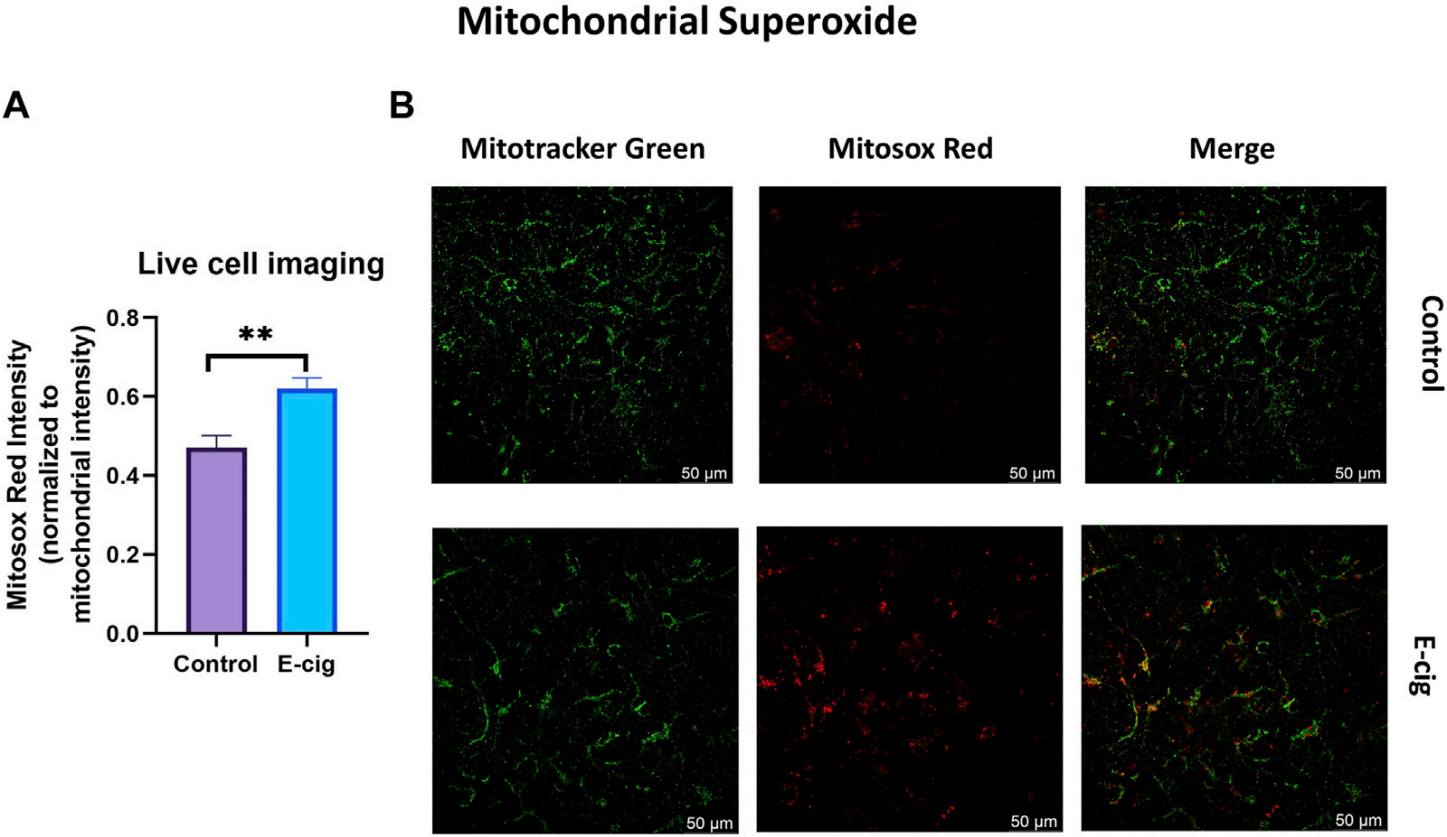
Mitochondrial superoxide was quantified in primary neuron using confocal imaging with Mitosox red (mitochondrial superoxide probe) and mitotracker green (mitochondria staining probe). Prenatally e-cig exposed primary neuron demonstrated significantly higher level of mitochondrial superoxide (*p* < 0.01) in live cell imaging **(A, B)**. Biological replicates, *n* = 3. Fluorescence intensity of mitosox red was normalized by the fluorescence intensity of mitotracker green.

TS and e-cig aerosol exposure have been identified to be associated with the upregulation of total cellular ROS and mitochondrial superoxide (Emma et al., 2022). Surprisingly, higher ROS and OS levels have been reported in prenatally tobacco-exposed neonates (Aydogan et al., 2013; Chan et al., 2016). Hence in our study, we wanted to observe the effect of maternal vaping on total cellular ROS and mitochondrial superoxide generation. In this study, we measured the total cellular ROS and mitochondrial superoxide in primary neurons with the appropriate probe CM-H_2_DCFDA and mitosox red, respectively, using confocal live cell imaging and microplate reading. We observed that primary neurons isolated from prenatally e-cig exposed pups have significantly higher cellular total ROS (*p* < 0.05) and mitochondrial superoxide (*p* < 0.01) compared to control (*n* = 3), as depicted in Figures 2, 3, respectively.

### *In-utero* e-cig exposure disrupts mitochondrial respiration in primary neurons

**FIGURE 4:**
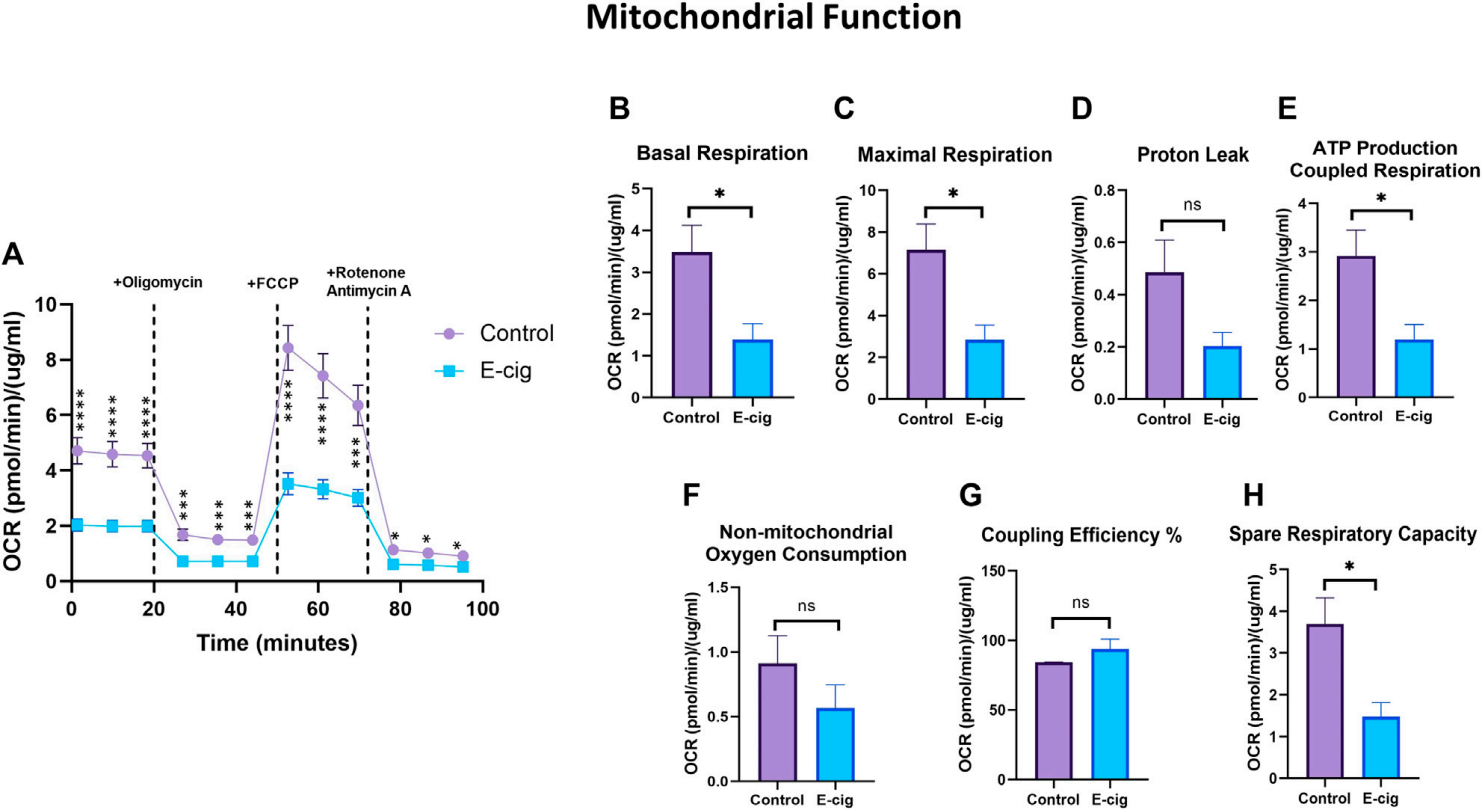
Assessment of mitochondrial function using XF24 extracellular flux analyzer. Prenatally e-cig exposed primary neuron had significantly lower **(A)** OCR compared to control at 1.38, 9.89,18.39,27, 35.51, 44.01, 52.62, 61.12, 69.62, 78.21, 86.71, 95.21 min. Significantly reduced level of OCR has been observed in **(B)** Basal respiration **(C)** Maximal respiration **(E)** ATP production coupled respiration **(H)** Spare respiratory capacity compared to control. However, no significant difference has been observed in oxygen consumption for **(D)** Proton leak **(F)** Non-mitochondrial oxygen consumption and **(G)** Coupling efficiency. Biological replicates, *n* = 3; data were normalized with respective protein concentration of the well. *p < 0.05, **p < 0.01, ***p < 0.001, ****p < 0.0001.

To explore the impact of maternal vaping on mitochondrial respiration in primary neurons, we measured the cellular OCR, which is related to mitochondrial respiratory chain function. There are several phases of the respiration cycle measured in the mitostress test. The respiration rate recorded at the initial phase of measurement is termed “Basal respiration,” which gives insights into the resting energetics of the cells. The addition of oligomycin inhibits mitochondrial ATP synthesis, thus decreasing the OCR by the mitochondrial respiratory chain. This represents the ATP produced by the mitochondria contributing to the energetic needs of the cell. The remaining OCR is used to balance mitochondrial proton leak but can also have a non-mitochondrial source which indicates other cellular processes using oxygen. The maximal OCR is attained by adding the uncoupler Carbonyl cyanide-4 (trifluoromethoxy) phenylhydrazone (FCCP), which mimics a physiological “energy demand” by stimulating the respiratory chain to operate at maximum capacity and causing the rapid oxidation of the substrates (sugars, fats, amino acids) to meet the metabolic challenge. Based on basal respiration, proton leak, and maximal respiration, it is possible to calculate the parameters describing (i) the respiration rate driving the synthesis of ATP under basal condition; (ii) coupling efficiency (which describes the fraction of basal respiration driving the ATP production versus proton leak and (iii) spare respiratory capacity indicating how close to maximal efficiency of the respiratory chain the cells are operating under basal conditions (Malinska et al., 2018). As shown in Figure 4A, prenatally e-cig-exposed primary neurons exhibited levels of OCR compared to the control. Significantly reduced levels of basal respiration (Figure 4B) and maximal respiration (Figure 4C) have been observed in the prenatally e-cig exposed group compared to the control (*p* < 0.05); however, proton leak was not significantly different (Figure 4D). Basal and maximal respirations and proton leaks can be used to evaluate OXPHOS function. In this study, we observed that the prenatally e-cig-treated group exhibited significantly reduced oxygen consumption levels coupled to ATP production (Figure 4E) and spare respiratory capacity (Figure 4H) when compared to the control (*p* < 0.05). However, non-mitochondrial oxygen consumption and coupling efficiency were not statistically different between the treated and control groups (see Figures 4F, G). The energy map depicted in Supplementary Figure S1 shows that prenatally e-cig exposed primary neurons are in a quiescent state, thus suggesting that these cells are not very energetic via either mitochondrial respiration or the glycolytic pathway.

### *In-utero* e-cig exposure downregulates antioxidative markers and upregulates pro-inflammatory modulators and tissue injury marker

**FIGURE 5:**
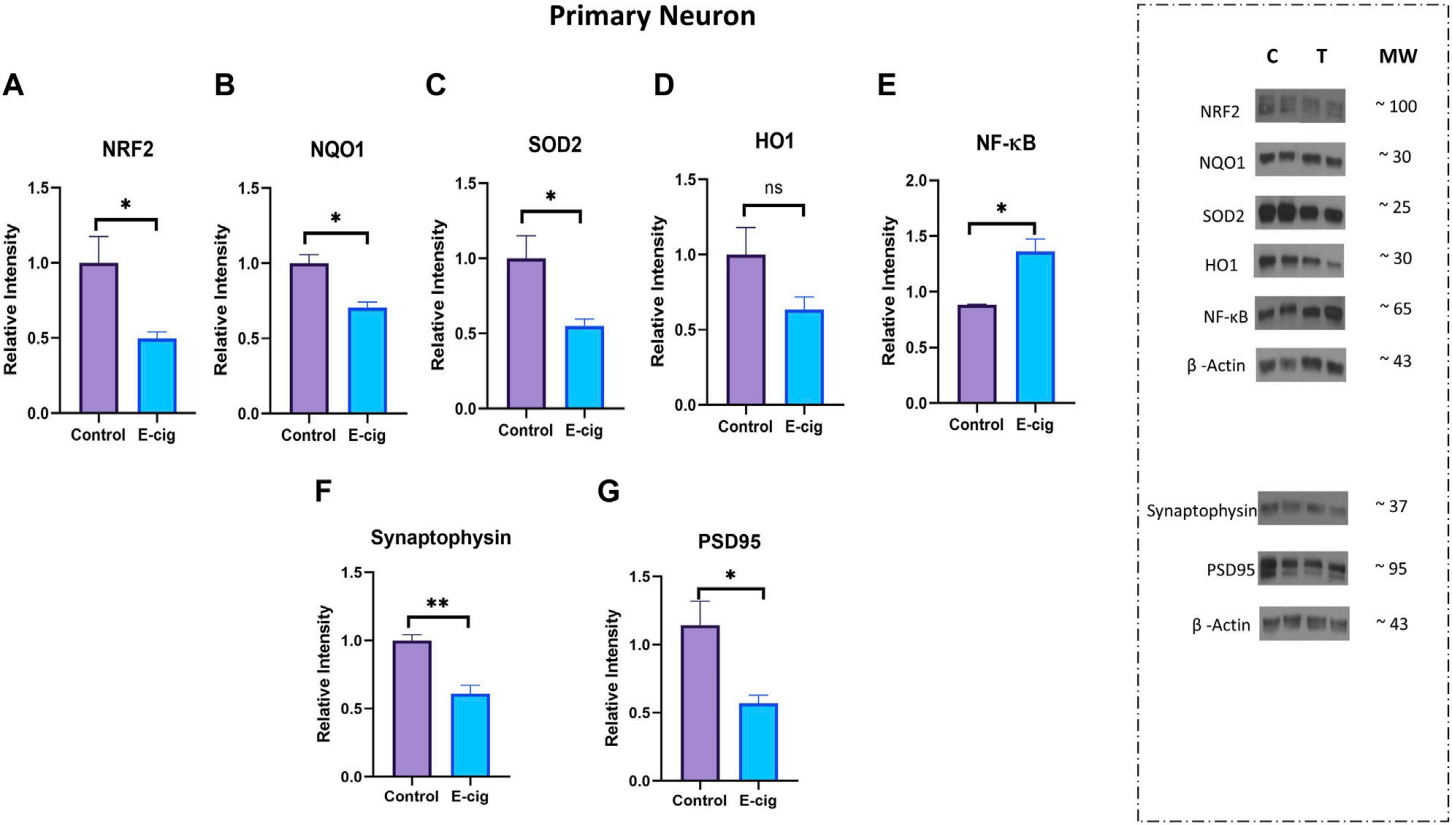
Measurement of anti-oxidative marker, pro-inflammatory modulator and synaptic marker in primary neuron by Western blot. Prenatally e-cig exposed offspring demonstrated significantly reduced expression of **(A)** NRF2 (*p* < 0.05), **(B)** NQO1 (*p* < 0.05), **(C)** SOD2 (*p* < 0.05), and increased expression of **(E)** NF-κ B (*p* < 0.05) in primary neuron compared to control. However, no significant difference was found in **(D)** HO-1 expression. Reduced expression of **(F)** presynaptic marker, synaptophysin (*p* < 0.01) and **(G)** postsynaptic marker, PSD95 (*p* < 0.05) have been observed in prenatally e-cig exposed primary neuron compared to control; Biological replicates, *n* = 3. Data were normalized to β-Actin. C, Control, T, Treatment.

**FIGURE 6:**
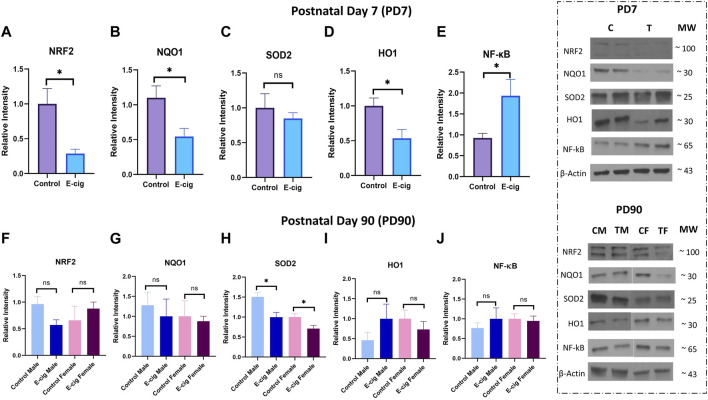
Measurement of anti-oxidative marker and pro-inflammatory modulator in postnatal brain at PD7 and PD90 by Western blot. Prenatally e-cig exposed offspring demonstrated significantly reduced expression of **(A)** NRF2 (*p* < 0.05), **(B)** NQO1 (*p* < 0.05), **(D)** HO1 (*p* < 0.05), and increased expression of **(E)** NF-κ B (*p* < 0.05) in postnatal brain at PD7 compared to control; *n* = 4. However, no significant difference was found in **(C)** SOD2 expression at PD7 between prenatally e-cig exposed offspring and control offspring. At PD90, no significant difference was found in expression of **(F)** NRF2, **(G)** NQO1, **(I)** HO1, **(J)** NF-κB; *n* = 4. However, prenatally e-cig exposed both male and female offspring demonstrated significantly reduced expression of **(H)** SOD2 compared to control; *n* = 4. Data were normalized to β-Actin. **C**, Control; T, Treatment; CM, Control Male; TM, Treatment Male; CF, Control Female; TF, Treatment Female.

**FIGURE 7:**
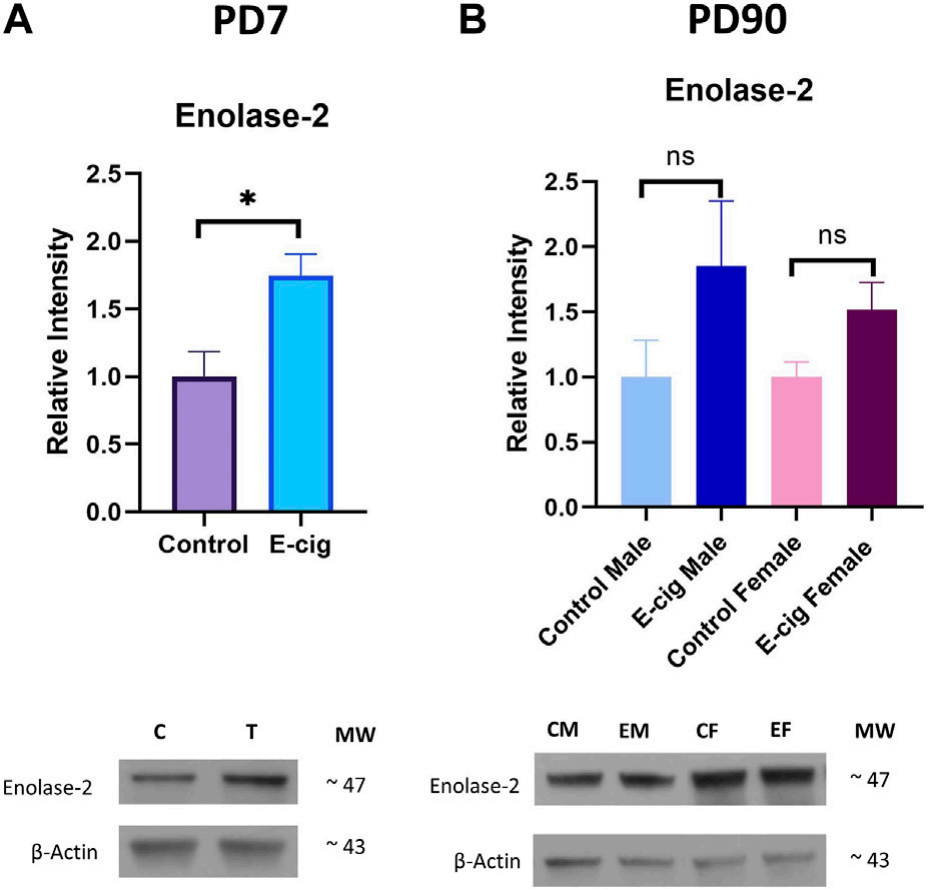
Measurement of neuronal injury marker Enolase-2 expression in postnatal brain at **(A)**. PD7 and **(B)**. PD90 by Western blot. Prenatally e-cig exposed offspring showed significantly higher expression level of Enolase-2 expression compared to control at PD7 (*p* < 0.05). However, no statistically significant difference has been observed in Enolase-2 expression at PD90 between prenatally e-cig exposed offspring and control offspring; Biological replicates, *n* = 4.

Studies have reported that nicotine, TS, and e-cig aerosol exposure are associated with the downregulation of antioxidative responses by reducing the activation of the NRF2-ARE pathway, which in turn downregulates the expression of its downstream detoxifying effector molecules NQO1, SOD, HO-1 and leads to NF-κB upregulation (Naha et al., 2017; Prasad et al., 2017; Li et al., 2019b; Sivandzade et al., 2019b). In our study, we assessed the effect of maternal vaping on the expression of antioxidative markers and pro-inflammatory modulators by Western blot analysis. Consistent with previously published reports, our data showed that maternal vaping significantly reduced the expression of NRF2 (*p* < 0.05), NQO1 (*p* < 0.05), and SOD2 (<0.05) while upregulating that of NF-κB (*p* < 0.05) in primary neuron isolated from prenatally e-cig exposed offspring compared to control (*n* = 3) (Figure 5). Similarly, in PD7, downregulation of NRF2 (*p* < 0.05), NQO1 (*p* < 0.05), HO1 (<0.05), and upregulation of NF-κB (*p* < 0.05) have been observed (*n* = 4) (Figure 6). However, we did not observe corresponding significant differences in either male or female PD90 offspring except for SOD2, which was significantly reduced (*p* < 0.05) in both genders (Figure 6). We also measured neuron specific tissue injury marker, Enolase-2 in postnatal brain and significantly higher level of Enolase-2 has been found in parentally e-cig exposed postnatal brain at PD7 but not in PD90 (Figure 7).

### *In-utero* e-cig exposure disrupts the expression of synaptic proteins in primary neurons

Since neuroinflammation is associated with synaptic markers loss, which may result in cognitive dysfunction and neurodegenerative diseases (Rao et al., 2012), we measured the expression of synaptic proteins, synaptophysin (pre-synaptic), and PSD95 (post-synaptic) in primary neurons. In this study, we observed significantly reduced expression of synaptophysin (*p* < 0.01) and PSD95 (*p* < 0.05) in prenatally e-cig-exposed primary neurons compared to control (*n* = 3), as depicted in Figures 5F, G.

### *In-utero* e-cig use upregulates pro-inflammatory cytokines’ levels and dysregulates anti-inflammatory cytokine levels in neurons and the brain

**FIGURE 8:**
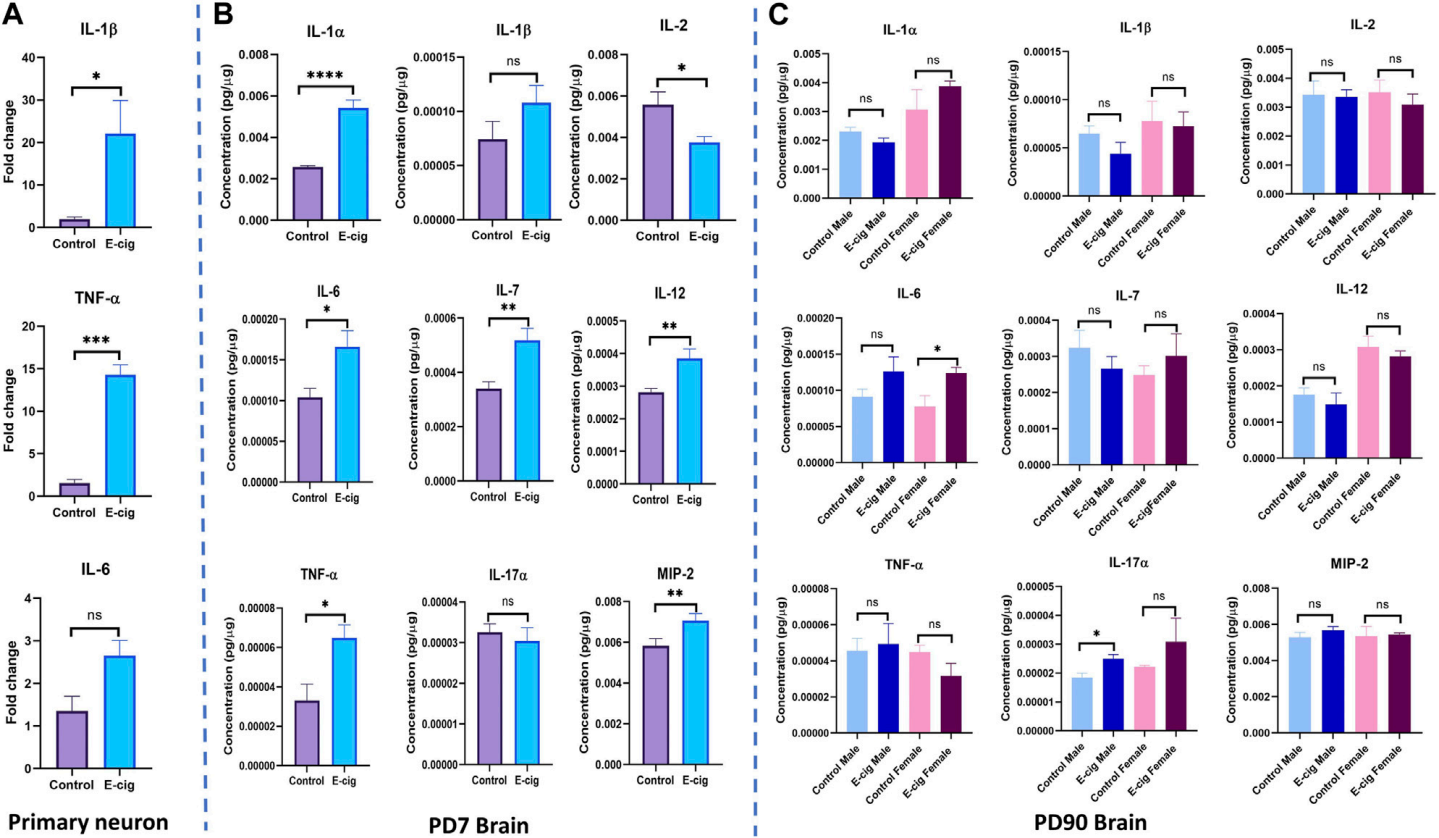
Measurement of pro-inflammatory cytokines in **(A)** Primary neuron **(B)** Postnatal brain at PD7 and **(C)** Postnatal brain at PD90. Prenatally e-cig exposed offspring demonstrated significantly high level of pro-inflammatory cytokines IL-1β (*p* < 0.05) and TNF-α (*p* < 0.001) in RT-PCR compared to control; Biological replicates, *n* = 3. However, no significant difference has been observed in IL-6 expression. Immunobead assay was performed to measure the pro-inflammatory cytokines in postnatal brain at **(B)** PD7 and **(C)** PD90. Prenatally e-cig exposed postnatal brain **(B)** at PD7, demonstrated significant upregulation of proinflammatory cytokines IL-1α (*p* < 0.0001), IL-2 (*p* < 0.05), IL-6 (*p* < 0.05), IL-7 (*p* < 0.01), IL-12 (*p* < 0.01), TNF-α (*p* < 0.05), MIP2 (*p* < 0.01). However, no significant difference has been observed in the expression of pro-inflammatory cytokines except for IL-6 and IL-17α **(C)** at PD90. Significantly higher expression of IL-6 (*p* < 0.05) was observed in prenatally e-cig exposed female offspring compared to control female offspring and IL-17α (*p* < 0.05) in prenatally e-cig exposed male offspring compared to control male offspring; *n* = 4. Data were normalized with respective protein concentration.

**FIGURE 9:**
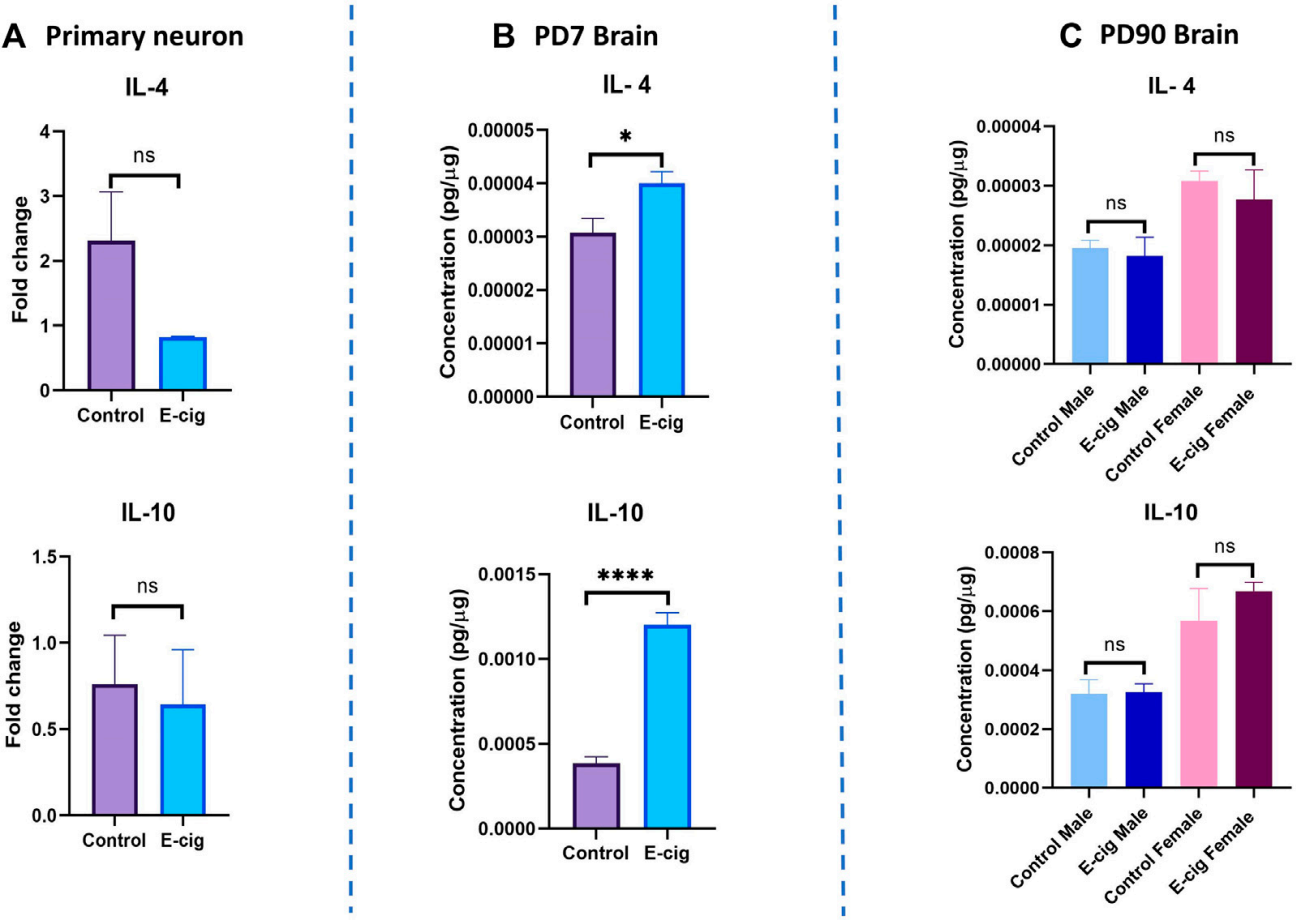
Measurement of anti-inflammatory cytokines in **(A)** Primary neuron **(B)** Postnatal brain at PD7 and **(C)** Postnatal brain at PD90. Prenatally e-cig exposed offspring demonstrated significantly high level of anti-inflammatory cytokines IL-4 (*p* < 0.05) and IL-10 (*p* < 0.0001) in **(B)** PD7 brain compared to control by immunobead assay; Biological replicates, *n* = 6. However, no significant difference has been observed in IL-4 and IL-10 expression in **(A)** Primary neuron by RT-PCR and **(C)** PD90 brain by immunobead assay; Biological replicates, *n* = 3 for primary neuron and *n* = 4 for PD90 brain. Immunobead assay data were normalized with respective protein concentration.

We also measured the level of the pro-inflammatory cytokines’ levels in primary neurons and brain lysate from PD7 and PD90 by RT-PCR and immunobead assay, respectively. In primary neurons, significantly higher levels of pro-inflammatory cytokines IL-1β (*p* < 0.05) and TNF-α (*p* < 0.001) have been found in RT-PCR assay with e-cig exposure compared to control (Figure 8) (*n* = 3). We also measured the cytokines’ levels at PD7 and PD90. At PD7, we observed significantly high levels of proinflammatory cytokines IL-1α (*p* < 0.0001), IL-6 (*p* < 0.05), IL-7 (*p* < 0.01), IL-12 (*p* < 0.01), TNF-α (*p* < 0.05) and MIP2 (*p* < 0.01) in prenatally e-cig exposed offspring brain (Figure 8). In contrast, at PD90, IL-6 has been found to be upregulated in prenatally e-cig exposed female offspring (*p* < 0.05) and IL-17α were upregulated in prenatally e-cig exposed male offspring (*p* < 0.05) (*n* = 4) (Figure 8). We also measured anti-inflammatory cytokines’ level (IL-4 and IL-10) in primary neuron by RT-PCR and in postnatal brain at PD7 and PD90. In prenatally e-cig exposed primary neurons, we did not find any significant difference in mRNA expression level of IL-4 and IL-10 compared to control (Figure 9), however significantly higher expression level of IL-4 (*p* < 0.05) and IL-10 (*p* < 0.001) have been observed in prenatally e-cig exposed postnatal brain at PD7 (Figure 9). We did not observe any significant difference at PD90 postnatal brain (Figure 9).

### *In-utero* e-cig exposure resulted in decreased body weight at PD7 and PD90

Consistent with our previously published literature (Archie et al., 2023), we also observed significantly reduced body weight in prenatally e-cig exposed both male and female offspring compared to control (Supplementary Figure S2). In our previous study, we also reported a significantly lower brain-to-body weight ratio in prenatally e-cig exposed offspring at PD7 but not in PD90 (Archie et al., 2023). The presence of nicotine and cotinine in offspring plasma was also confirmed (Archie et al., 2023).

## Discussion

Cellular homeostasis depends on the regulated levels of ROS. Low or moderate levels of ROS can act as signaling molecules, which are crucial to maintaining normal cellular function. On the contrary, uncontrolled ROS generation causes oxidative damage contributing to cell dysfunction and damage (Chen et al., 2018b). Studies have shown that physiological levels of ROS can play pivotal roles in cerebral vasculature health (De Silva and Miller, 2016). ROS can contribute to the regulation of brain perfusion through their action in vascular tone control and vasodilation (Carvalho and Moreira, 2018).

Moreover, under normal physiological conditions, ROS induces mitogenic response and immune defense mechanisms and regulate signal transduction, blood flow, and effectors of preconditioning mechanisms. However, when ROS concentrations cross a certain threshold, they elicit an increase in blood flow resistance, decreased nitric oxide (•NO) bioavailability, reduced vasodilatation and immune response, increased apoptosis, and the downregulation of endothelial nitric oxide synthase (eNOS), thus promoting the onset of pathological conditions. The delicate balance of ROS levels is regulated by cellular antioxidant systems such as glutathione (GSH), GPX1, GSR, NQO1, SOD, CAT, NRF2 and antioxidant response element (ARE) (Carvalho and Moreira, 2018).

The neurovascular unit (NVU) contains neurons, astrocytes, pericytes, microglia, and endothelial cells and is equipped with a powerful antioxidant defense system encompassing a plethora of antioxidative enzymes (Tayarani et al., 1987; Halliwell, 2001; Sivandzade et al., 2019b). Particularly, GSH has been shown to play a crucial role in maintaining BBB integrity (Agarwal and Shukla, 1999). Moreover, NRF2 seems to play a major defense role in the brain by regulating the expression of antioxidant enzymes (Carvalho and Moreira, 2018; Sivandzade et al., 2019b), controlling microglial dynamics (Rojo et al., 2010), protecting astrocytes and neurons from toxic insults (Lee et al., 2003; Vargas and Johnson, 2009), regulating the expression of antioxidant enzymes (Shah et al., 2007; Yan et al., 2008) and inducing secondary defense proteins via interaction with the ARE in the promoter region of target genes (Carvalho and Moreira, 2018). Additionally, NRF2 can downregulate the pro-inflammatory modulator NF-κB to maintain cellular physiological ROS levels (Sivandzade et al., 2019b). NF-κB is a redox-regulated transcription factor involved in the modulation of inflammation, immune function, cellular growth, and apoptosis. OS can activate IκB kinase (IKK), which phosphorylates the NF-κB inhibitor leading to polyubiquitination mediated proteasomal degradation of IκB and the release of NF-κB. NF-κB then migrates into the nucleus, binds with its corresponding DNA responsive elements (the κ region of genome) and along with other coactivators, promotes the transcription of proinflammatory mediators and cytokines such as TNFα, IL-1α/β (Sivandzade et al., 2019b). Studies have demonstrated an interplay between NRF2 and NF-κB signaling pathways under stress and pathologic condition (Wardyn et al., 2015). Downregulation of NRF2 increases inflammation, whereas its upregulation decreases pro-inflammatory and immune responses transcriptionally regulated by NF-ĸB. In summary, NRF2 and NF-κB are regulated antagonistically. If NRF2 predominates, it decreases inflammation and oxidative stress through activation of antioxidant enzymes; however, when NF-κB predominates, it promotes the secretion of pro-inflammatory mediators, suppresses NRF2 activity (thus preventing the transcription of the ARE genes), and sustains/exacerbates OS conditions (Helou et al., 2019).

Certain exogenous stimuli, such as TS and nicotine exposure, have been shown to affect NRF2- NF-κB signaling pathway resulting in OS and inflammation (Sivandzade et al., 2019b). Nicotine not only induces OS, apoptosis, and inflammation in brain cells (Oliveira-da-Silva et al., 2009; Benowitz, 2010; Cardinale et al., 2012) but also impacts BBB integrity, promotes cerebrovascular dysfunction (Archie et al., 2022), and exacerbates pathological behavioral outcomes in mice (Shim et al., 2008). Several studies have shown a correlation between TS/nicotine and lower mitochondrial respiration. Dose-dependent inhibitory effects of nicotine on mitochondrial oxygen consumption have been observed in rat brains (Cormier et al., 2001). Nicotine inhibits mitochondrial oxygen consumption by directly influencing the respiratory chain complexes. A [^3^H]-nicotine binding assay demonstrated that nicotine binds to complex I and inhibits its NADH-ubiquinone reductase activity and results in inhibition of electrons flowing from NADH to complex I (Cormier et al., 2001), which is linked to a detectable decrease in oxygen consumption by mitochondria (Malińska et al., 2019). Even, maternal smoking has been identified as a risk factor for poor postnatal health, including increased OS and pro-inflammatory cytokines, mitochondrial dysfunction (Chan et al., 2016; Lei et al., 2018; Ji et al., 2021), reduced antioxidative enzymes as well as glutathione content (Lei et al., 2018) which may result in several cerebrovascular dysfunction and neurodegenerative diseases (Sivandzade et al., 2019a) in neonates.

Maternal smoking has also been associated with mitochondrial dysfunction, including mitochondrial ultrastructure changes, mitochondrial swelling, reduced ATP generation, loss of mitochondrial membrane potential, and increased mitochondrial DNA copy number (Lei et al., 2018). Interestingly, recently introduced e-cig products, considered a healthier alternative to TS, have also been associated with the upregulation of OS, proinflammatory cytokines, and mitochondrial dysfunction leading to inflammation (Muthumalage et al., 2018; Espinoza-Derout et al., 2019; Han et al., 2021; Ma et al., 2021; Kanithi et al., 2022). E-cig use also disrupts mitochondrial respiration and causes mitochondrial damage (Kanithi et al., 2022) However, there are a limited number of preclinical and clinical studies assessing the role of maternal vaping on postnatal neuroimmune and mitochondrial function. Hence, we evaluated the impact of *in utero* e-cig use on neonatal OS, cytokines, mitochondrial function, and antioxidative mechanism in this study.

Herein, pregnant CD1 mice were exposed to Blu e-cig products containing 2.4% nicotine from gestational day E5 to postnatal day PD7. In between, gestational day E16/17, we isolated primary neurons from pups from randomly selected mothers. Our goal was to evaluate the impact of e-cig on postnatal neurons since they are quite sensitive to even moderate levels of OS compared to astrocytes (Vargas and Johnson, 2009). We measured the total cellular ROS and mitochondrial superoxide by live cell imaging. In both cases, we observed significantly higher levels of total ROS and mitochondrial superoxide in prenatally e-cig-exposed primary neurons. Next, we evaluated the expression of antioxidative enzymes and pro-inflammatory modulators, and in prenatally e-cig exposed primary neurons, we observed significantly lower expression levels of NRF2, NQO1, SOD2, and higher levels of NF-ĸB compared to controls, thus indicating that *in utero* e-cig exposure disrupts NRF2- NF-ĸB pathway resulting in the downregulation of the antioxidant defenses and the upregulation of ROS. These findings are consistent with previously published reports showing that nicotine and TS are responsible for disrupting the NRF2- NF-ĸB interplay leading to increased ROS production and OS (Garbin et al., 2009; Prasad et al., 2019). In this study, we also observed significantly reduced expression of NRF2, NQO1, HO-1, and higher levels of NF-ĸB in PD7 offspring, suggesting the persistent impact of maternal vaping on neonatal neuroimmune function. At PD90, SOD2 levels were significantly downregulated in both male and female offspring of mice chronically exposed to e-cig vape compared to controls. However, no significant differences were observed regarding NRF2, NQO1, HO-1, and NF-ĸB expression between controls and *in utero* e-cig-exposed offspring. These data suggest that the endogenous antioxidative mechanisms are perhaps still compromised in the long term at adulthood.

One of the interesting things about NRF2 is that it has deleterious effects on regulation of transporters such as multidrug resistance proteins (Mrps), which are known to be highly expressed at the neurovascular unit. Nrf2 has been found to induce expression of Mrp1, Mrp2, and Mrp4 and the genes encoding these proteins (i.e., Abcc1, Abcc2, Abcc4) at the BBB is a critical determinant in controlling delivery of therapeutic agents to the brain (Ibbotson et al., 2017). GSH is a known Mrp transporter substrate and changes in Mrp protein levels due to altered Nrf2 signaling can lead to significant modulation of CNS GSH levels. GSH is essential in maintaining cellular redox balance and antioxidant defense mechanism in the brain. Upregulation of Mrps due to OS leads to enhanced cellular efflux of GSH (Ronaldson and Bendayan, 2008) resulting in reduced endothelial cell concentrations of GSH which may increase the potential for cell injury and death. Therefore, it would be interesting to see if the altered NRF2 level dysregulates the expression of Mrps in response to e-cig mediated OS in postnatal brain in future experimental studies.

Interestingly, we also observed that prenatally e-cig-exposed primary neurons demonstrated a disruption in mitochondrial function. A significantly reduced level of OCR in basal and maximal respirations in prenatally e-cig-exposed primary neurons suggests that maternal e-cig exposure can disrupt neuronal bioenergetic metabolism. Since neurons rely on oxidative metabolism to meet their high energy demands (Bélanger et al., 2011), disruption of mitochondrial function can negatively impact neuronal viability and activity. Nicotine and TS can disrupt the mitochondrial chain function and imbalance the mitochondrial dynamics (Meng et al., 2021; Kanithi et al., 2022). Nicotine/TS are both ROS production promoters and the resulting increase in ROS generation can negatively impair the efficiency of mitochondrial oxygen utilization (Zhao et al., 2020). Therefore, increased ROS generation and consequent impaired mitochondrial activity in postnatal primary neurons of the offspring of mice chronically exposed to e-cig can result in neonatal brain insults at a later stage. However, the absence of significant difference in proton leak between controls and the offspring of e-cig-exposed mice suggests that *in utero* e-cig exposure does not affect neurons’ mitochondrial inner membrane integrity. The energy map provided in Supplementary Figure S1 also clearly shows the quiescent state of prenatally e-cig-exposed primary neurons suggesting that these cells are not using either mitochondrial respiration or glycolytic pathways compared to their control counterparts. This is alarming since neurons predominantly use mitochondrial respiration under normal physiological conditions to meet their high energy demands (Bélanger et al., 2011).

Our study also showed that the neurons from prenatally e-cig-exposed mice exhibited a significantly lower ATP production level than controls which is consistent with our previous findings. We also observed a lower level of non-mitochondrial oxygen consumption in prenatally e-cig exposed primary neurons compared to control, although not significant which is consistent with the energy map provided (Supplementary Figure S1). As expected from the energy map, primary neurons from prenatally e-cig exposed pups were not using non-mitochondrial oxygen sources or the glycolytic pathway for meeting energy demand and non-mitochondrial oxygen consumption data also showed lower values in prenatally e-cig exposed primary neuron.

OS and mitochondrial dysfunction are associated with increased proinflammatory cytokines (Elmarakby and Sullivan, 2012; Hoffmann et al., 2019). Consistent with our findings, we also observed significantly higher expression of pro-inflammatory cytokines in prenatally e-cig-exposed primary neurons and postnatal brains. At PD7, we observed significant higher expression of several pro-inflammatory cytokines including IL-1α, IL-2, IL-6, IL-7, IL-12, TNF-α, MIP-2 in prenatally e-cig exposed postnatal brain; however, at PD90, we only observed statistically higher expression of IL-6 in prenatally e-cig exposed female offspring and IL-17α in prenatally ex-cig exposed male offspring indicating the sexual dimorphism in immune responses and cytokine production (Klein and Flanagan, 2016). Although there was higher expression of IL-6 in prenatally e-cig exposed male offspring and IL-17α in prenatally e-cig exposed female offspring, however the difference was not statistically significant. Therefore, dedicated future studies on the effects of maternal vaping on postnatal cytokine production needs to be conducted to explore more on sexual dimorphism.

Usually, enhanced expression of pro-inflammatory cytokines triggers a concurrent upregulation of anti-inflammatory cytokines and in our study, we also observed significantly higher expression of IL-4 and IL-10 in postnatal brain at PD7. The relationship between proinflammatory cytokines and anti-inflammatory cytokines is complex, and their interplay is tightly regulated to maintain immune homeostasis. In certain situations, proinflammatory cytokines can induce the production of anti-inflammatory cytokines as part of a negative feedback mechanism to combat the immune response and prevent excessive inflammation. For example, IL-10 is an important anti-inflammatory cytokine that can be induced by various proinflammatory cytokines, including TNF-α and IL-6. IL-10 acts by inhibiting the production and activity of proinflammatory cytokines, thereby exerting anti-inflammatory effects (Shmarina et al., 2001; Jancálek et al., 2010; Sapan et al., 2016). In our study, we also observed an increased level of pro-inflammatory IL-6 and TNF- α and anti-inflammatory cytokines IL-4 and IL-10 demonstrating the concurrent immune reaction in response to inflammation induced by maternal e-cig exposure.

We also observed significantly reduced expression of synaptophysin (*p* < 0.01) (Figure 5), which is a specific and sensitive marker for synaptic terminals in primary neurons from prenatally e-cig-exposed pups. Synaptophysin is the major protein of the synaptic membrane and may play a crucial role in synaptic vesicle exocytosis, e.g., in neurotransmitter release (Hajjar et al., 2013). As synaptophysin and other synaptic vesicle proteins are associated with cellular plasticity underlying learning (Janz et al., 1999), downregulation of synaptophysin could be associated with loss of cognitive function (Hajjar et al., 2013) and several neurodegenerative diseases (Bai and Strong, 2014), which suggests additional challenges to neonates with respect to new synapse formation due to maternal vaping. We also observed a significant decrease in PSD95 expression, which is a post-synaptic marker associated with learning and memory function. Studies have shown that PSD95 is an important synaptic protein which promotes synapse maturation (Barrow and McAllister, 2013), regulates synaptic plasticity (Dore et al., 2021) as well as supports the stability of hippocampus-dependent memory (Fitzgerald et al., 2015). Our previous research reported that maternal e-cig exposure is associated with deteriorated motor, learning, and memory function, which is consistent with this new finding (Archie et al., 2023). As excessive ROS generation at the site of inflammation causes endothelial dysfunction and tissue injury (Mittal et al., 2014), therefore we wanted to evaluate the expression level of neuronal injury marker, Enolase-2 in postnatal brain at PD7 and PD90. A significant upregulation of Enolase-2 at PD7 indicates that enhanced ROS production due to *in utero* e-cig exposure may result in tissue injury in postnatal brain. More investigations need to be conducted focusing on maternal e-cig exposure mediated tissue injury in postnatal brain. Therefore, in future we would like to evaluate the impact of maternal e-cig use on postnatal ischemic stroke injury.

## Conclusion

E-cigs have been proposed as an effective smoking cessation tool and tobacco replacement therapy. However, the potential health impact of vaping (instead of smoking) has not been fully evaluated. This major knowledge gap is even more concerning for pregnant women and neonates, considering that e-cigarette use during pregnancy is perceived by some consumers as less harmful (for both the mother and the fetus) than conventional tobacco products, even though no data are available in the literature. Hence, our current study aimed to elucidate the potential impact of e-cig aerosol exposure in mice during pregnancy on neonatal health. Our study indicated that e-cig exposure during pregnancy could promote OS, inflammation, and mitochondrial dysfunction in the postnatal brain, ultimately compromising the viability and integrity of the brain microvascular system and increasing the susceptibility to future neurodegenerative diseases in exposed neonates. Our findings highlight the potential for valid safety concern of e-cig use during pregnancy, which could be detrimental to neonatal brain development. These preclinical findings should raise concern for those who believe the use of e-cig is less harmful than traditional tobacco cigarettes for pregnant smokers.

## Data Availability

The original contributions presented in the study are included in the article/Supplementary Material, further inquiries can be directed to the corresponding author.

## Supplementary material

SUPPLEMENTARY FIGURE S1 Representative figure of energy map for primary neuron. Prenatally e cig exposed primary neuron demonstrated quiescent state which means the primary neuron isolated from prenatally e-cig exposed pups are not very energetic in either metabolic pathway (mitochondrial respiration or glycolytic). Primary neuron from control group are mostly using mitochondrial respiration.

SUPPLEMENTARY FIGURE S2 Measurement of postnatal weight at PD7 and PD90. Prenatally e-cig exposed both male and female offspring demonstrated significantly reduced body weight at PD7 (*p* < 0.0001) and PD90 (*p* <0.001) for male and (*p* < 0.0001) for female); Biological replicates, *n*=20–40.

## Abbreviations

ARE, Antioxidant response element; BBB, Blood-brain barrier; CAT, Catalase; E-cig, Electronic cigarette; E5, Gestational day 5; E16/17, Gestational day 16/17; eNOS, Endothelial nitric oxide synthase; FCCP, Carbonyl cyanide-4 (trifluoromethoxy) phenylhydrazone; GST, Glutathione transferase; GPX1, Glutathione peroxidase; HO1, Heme oxygenase; IKK, IκB kinase; IL, Interleukin; LBW, Low birth weight; NRF2, nuclear factor erythroid 2-related factor 2; NQO1, NAD(P)H quinone oxidoreductase 1; NF-κb, Nuclear factor kappa B; NO, Nitric oxide; NVU, Neurovascular unit; OS, Oxidative stress; OXPHOS, Oxidative phosphorylation; OCR, Oxygen consumption rate; PD7, Postnatal day 7; PD90, Postnatal day 90; ROS, Reactive oxygen species; SOD, Superoxide dismutase; SIDS, Sudden infant death syndrome; TS, Tobacco smoke.
