# Supplementary figures and images for: Impact of in-utero electronic cigarette exposure on neonatal neuroinflammation, oxidative stress and mitochondrial function
Source: Front Pharmacol. 2023 Aug 24;14:1227145. doi: 10.3389/fphar.2023.1227145 (PMC10484598; doi:10.3389/fphar.2023.1227145)

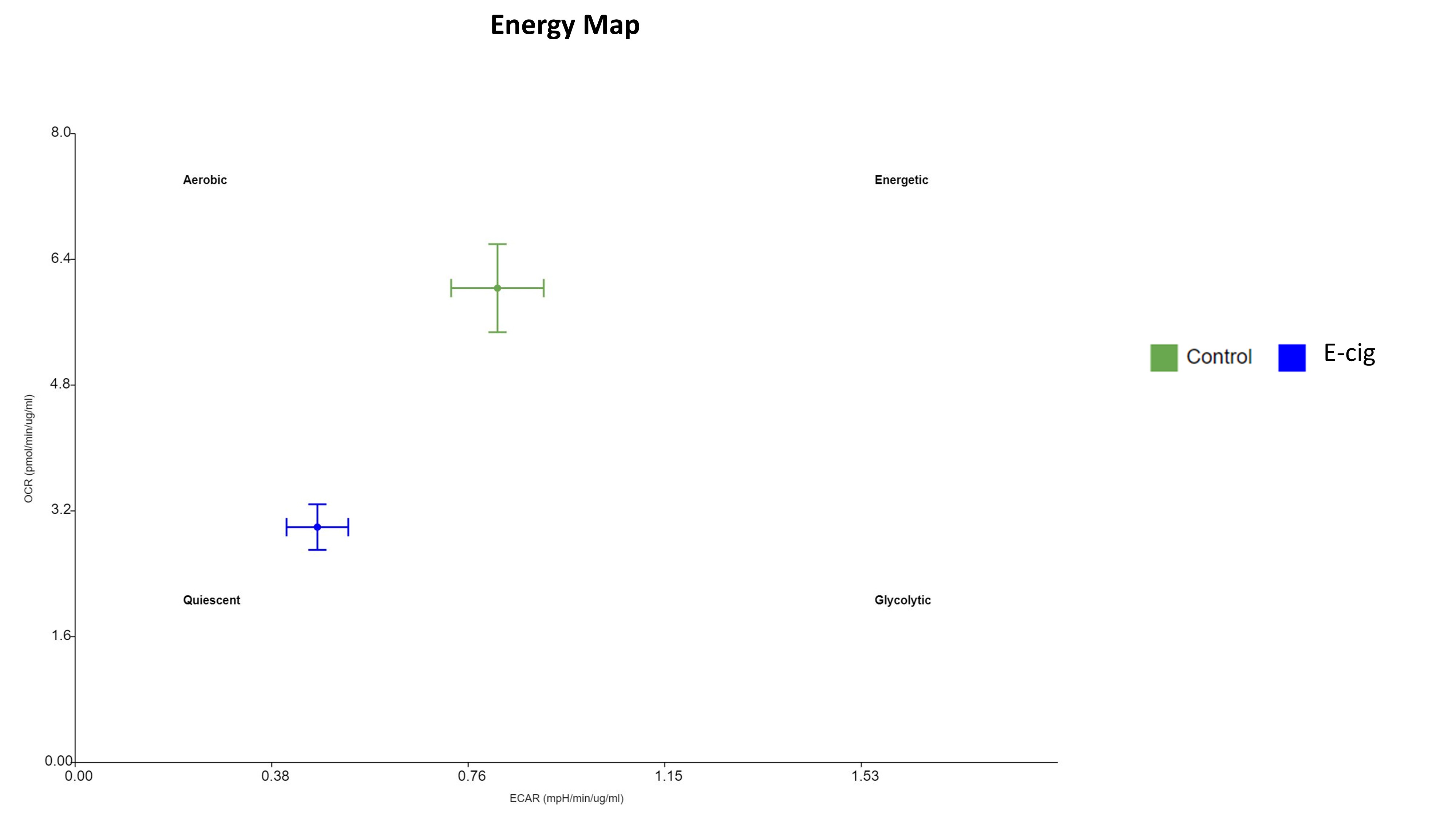

Supplement: Supplementary file 1 [file Image1.JPEG]

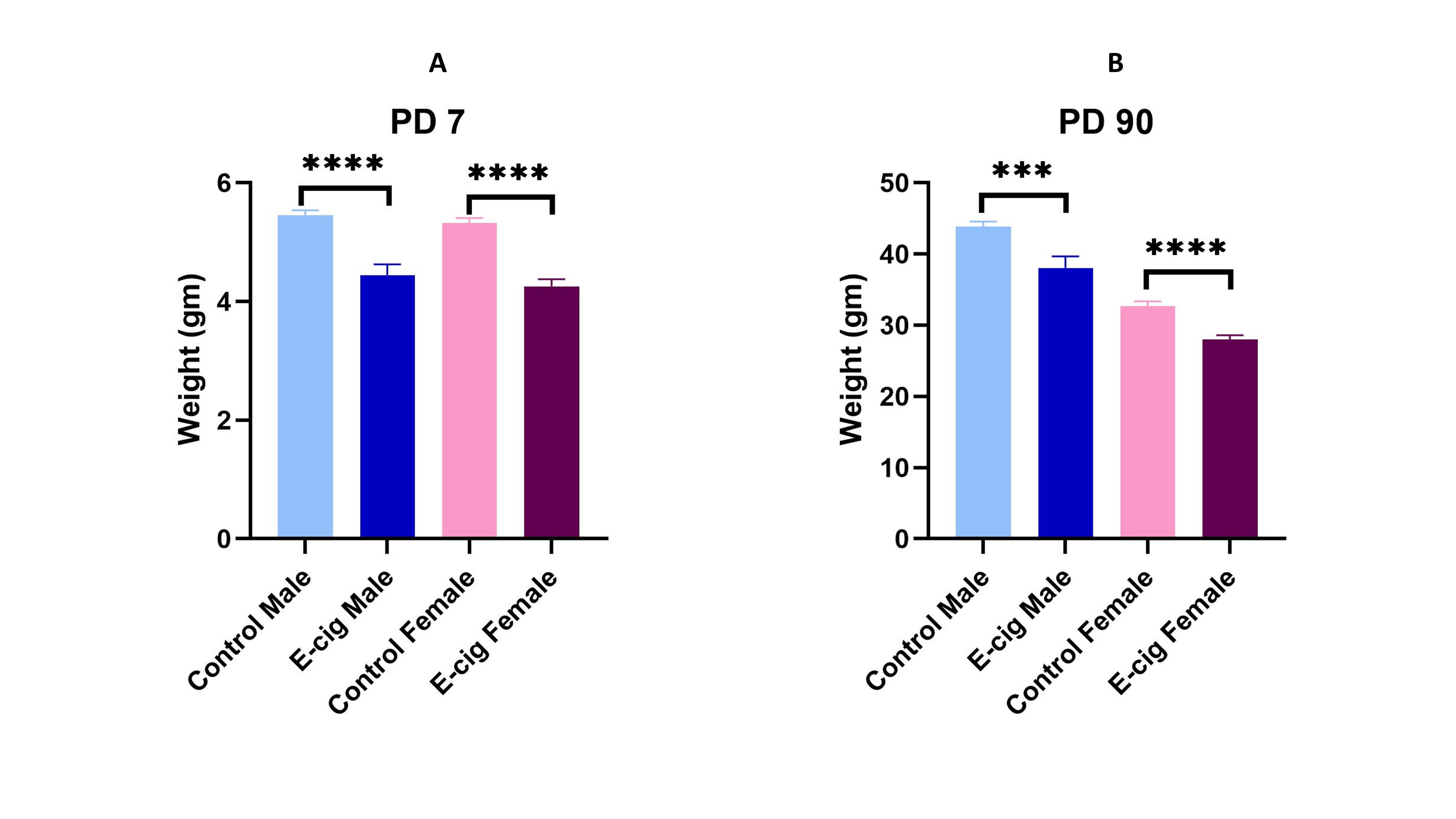

Supplement: Supplementary file 2 [file Image2.JPEG]
